# Case Report: From lung to peritoneum: genomic and immunologic insights into a rare dual-site *Corynebacterium striatum* infection

**DOI:** 10.3389/fimmu.2025.1685509

**Published:** 2025-10-15

**Authors:** Ying Tang, Fei Yan, Xincheng Huang, Qin Tang, Guolin Song

**Affiliations:** ^1^ Department of Clinical Laboratory, The Fourth People’s Hospital of Chengdu, Chengdu, China; ^2^ Department of Clinical Laboratory, The Clinical Hospital of Chengdu Brain Science Institute, MOE Key Lab for Neuroinformation, University of Electronic Science and Technology of China, Chengdu, China

**Keywords:** Corynebacterium striatum, multidrug resistance, whole-genome sequencing, intra-abdominal infection, immunocompromised host

## Abstract

*Corynebacterium striatum* is an emerging multidrug-resistant pathogen in immunocompromised patients. We report a 75-year-old male with paranoid schizophrenia on long-term clozapine who developed sepsis after gastric perforation repair. Within two days, genetically highly homologous *Corynebacterium striatum* strains were isolated from bronchoalveolar lavage fluid and ascitic fluid. Whole-genome sequencing revealed only 54 Single Nucleotide Polymorphisms (SNPs) differences, identical *ermX*-mediated resistance, and susceptibility to vancomycin and linezolid. Targeted therapy combined with drainage led to full recovery. This case highlights the potential for multi-site clonal infection via ICU environmental spread or endogenous translocation in the setting of postoperative immune suppression and invasive devices. Early multi-site sampling, molecular typing, and tailored antimicrobial therapy are essential, particularly in psychiatric patients requiring coordinated infection and psychiatric care.

## 1 Introduction

*Corynebacterium striatum* is a Gram-positive, facultatively anaerobic, non–spore-forming bacillus that was once largely regarded as a commensal colonizer of skin and mucous membranes, or merely an occasional contaminant (1). In recent years, however, reports of *Corynebacterium striatum* as an opportunistic pathogen have risen sharply, coinciding with the growing use of invasive procedures, prolonged administration of broad-spectrum antibiotics, and the increasing number of ICU patients (2). It has been implicated in infections of the respiratory tract, bloodstream, bone tissue, and prosthetic devices, and is frequently characterized by a multidrug-resistant (MDR) profile (2).

Its remarkable resistance and persistence are closely linked to biofilm formation, evasion of innate immunity, and selective pressures within the hospital environment (3). In hosts with impaired immune function, the pathogenic potential of *Corynebacterium striatum* becomes markedly amplified. Advanced age, postoperative stress, malnutrition, indwelling catheters, and extended exposure to broad-spectrum antimicrobials can all weaken neutrophil function and compromise mucosal barriers. Once these defenses are breached, the organism can invade sterile compartments such as ascitic fluid or the distal lower respiratory tract, where it may activate inflammatory pathways via TLR2, triggering IL-6 release; dynamic changes in procalcitonin (PCT) and C-reactive protein (CRP) levels can serve as indicators of inflammatory burden and therapeutic response (4).

Cases of *Corynebacterium striatum* causing ascitic or intra-abdominal infection are rare, and it is even more unusual to isolate genetically homologous strains from different anatomical sites within a short interval. In this report, we describe a postoperative elderly patient from whom *Corynebacterium striatum* (Cstriatum5) was first isolated from bronchoalveolar lavage fluid, followed the next day by recovery of a homologous strain (Cstriatum4) from ascitic fluid. By integrating clinical course, temporal trends in infection markers, and antimicrobial susceptibility profiles—supported by whole-genome sequencing for homology and resistance gene analysis—we aim to demonstrate that *Corynebacterium striatum* can cause true multi-site infections in immunocompromised hosts, propose an immuno-inflammatory biomarker–based approach for therapeutic evaluation, and provide evidence-based guidance for infection management and hospital infection control in vulnerable patient populations.

## 2 Narrative

### 2.1 Patient information

The patient was a 75-year-old male with a 46-year history of paranoid schizophrenia, maintained on long-term oral clozapine therapy. He was recently admitted for a gastric perforation, for which he underwent laparoscopic repair at an outside hospital. Postoperatively, he experienced persistent low-grade fever. His medical history was negative for diabetes mellitus, chronic obstructive pulmonary disease, or other major comorbidities; however, prolonged psychiatric medication use and poor preoperative nutritional status (serum albumin 27.9 g/L) suggested impaired immune function and increased susceptibility to infection.

### 2.2 Clinical findings

On postoperative day 8, the patient was transferred to the ICU due to acute exacerbation of psychiatric symptoms accompanied by abdominal pain, fever, and hemodynamic instability, consistent with acute peritonitis and sepsis. Upon ICU admission, levels of PCT, IL-6, WBC, and NEU% were markedly elevated, indicating a pronounced systemic inflammatory response. Both the respiratory tract and abdominal cavity were considered potential foci of infection.

### 2.3 Diagnostic assessment

**Figure 1:**
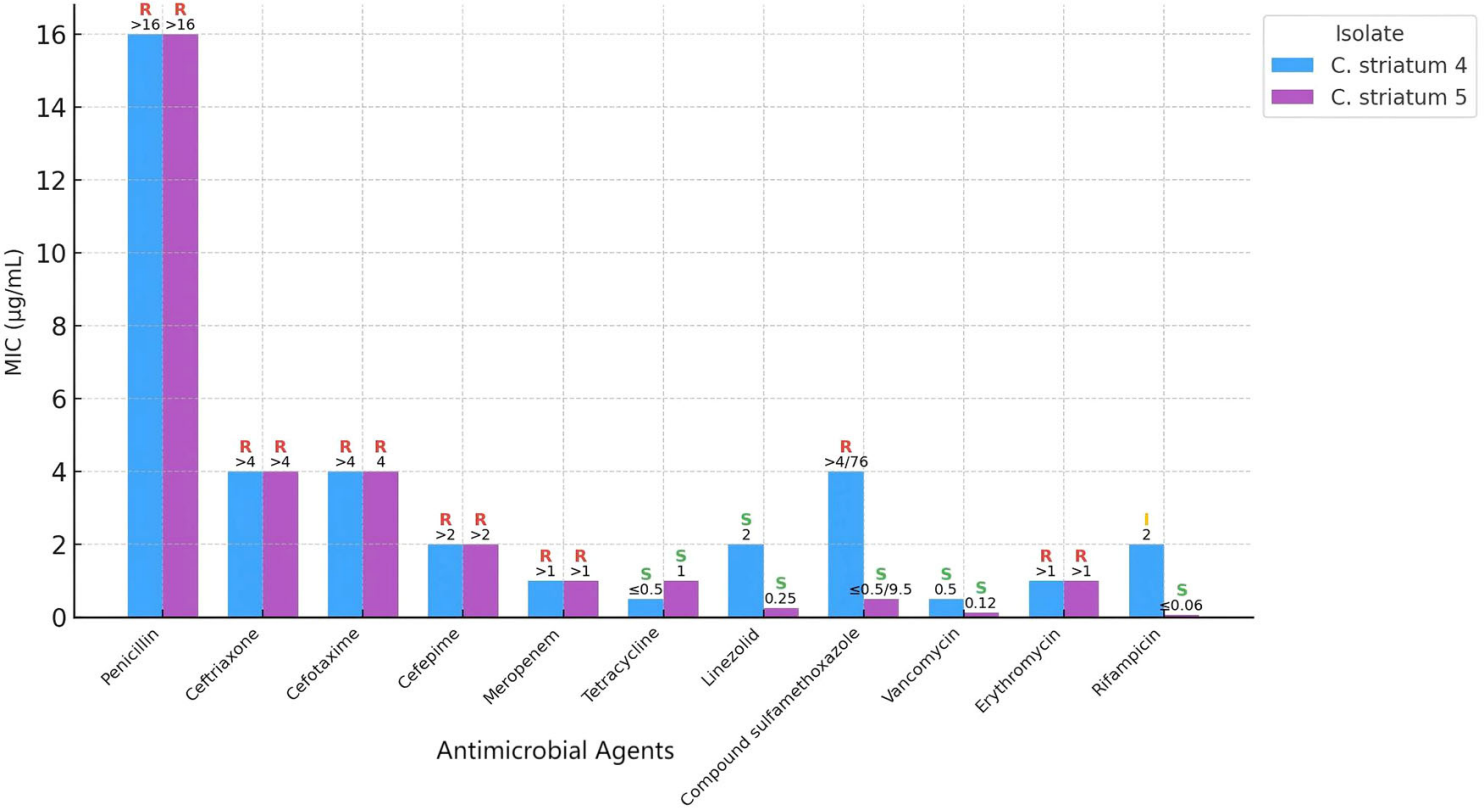
Comparative antimicrobial susceptibility profiles of *Corynebacterium striatum* strains Cstriatum4 and Cstriatum5.

On postoperative day 10, quantitative culture of the bronchoalveolar lavage fluid (BALF) yielded >10^4 CFU/mL. Gram staining demonstrated Gram-positive rods at 4+ and numerous leukocytes, consistent with an inflammatory process. MALDI-TOF MS (Autobio MS1000 system, Autobio Diagnostics, Zhengzhou, China) identified the isolate as *Corynebacterium striatum* (designated Cstriatum5).On postoperative day 11, culture of ascitic fluid once again yielded *Corynebacterium striatum* (designated Cstriatum4). Gram stain microscopy of the fluid revealed abundant leukocytes together with Gram-positive rods, further supporting the diagnosis of peritoneal infection. Antimicrobial susceptibility testing (performed according to CLSI M45 (5) guidelines) indicated multidrug resistance, with susceptibility retained only to vancomycin and linezolid (see **Figure 1**).

Numbers indicate minimum inhibitory concentration (MIC) values for each antimicrobial agent tested. S, I, and R represent susceptible, intermediate, and resistant categories, respectively, according to CLSI breakpoints. Although the two strains are genetically homologous, their antimicrobial susceptibility results are largely consistent, demonstrating similar resistance and sensitivity patterns across the tested agents.

**Figure 2:**
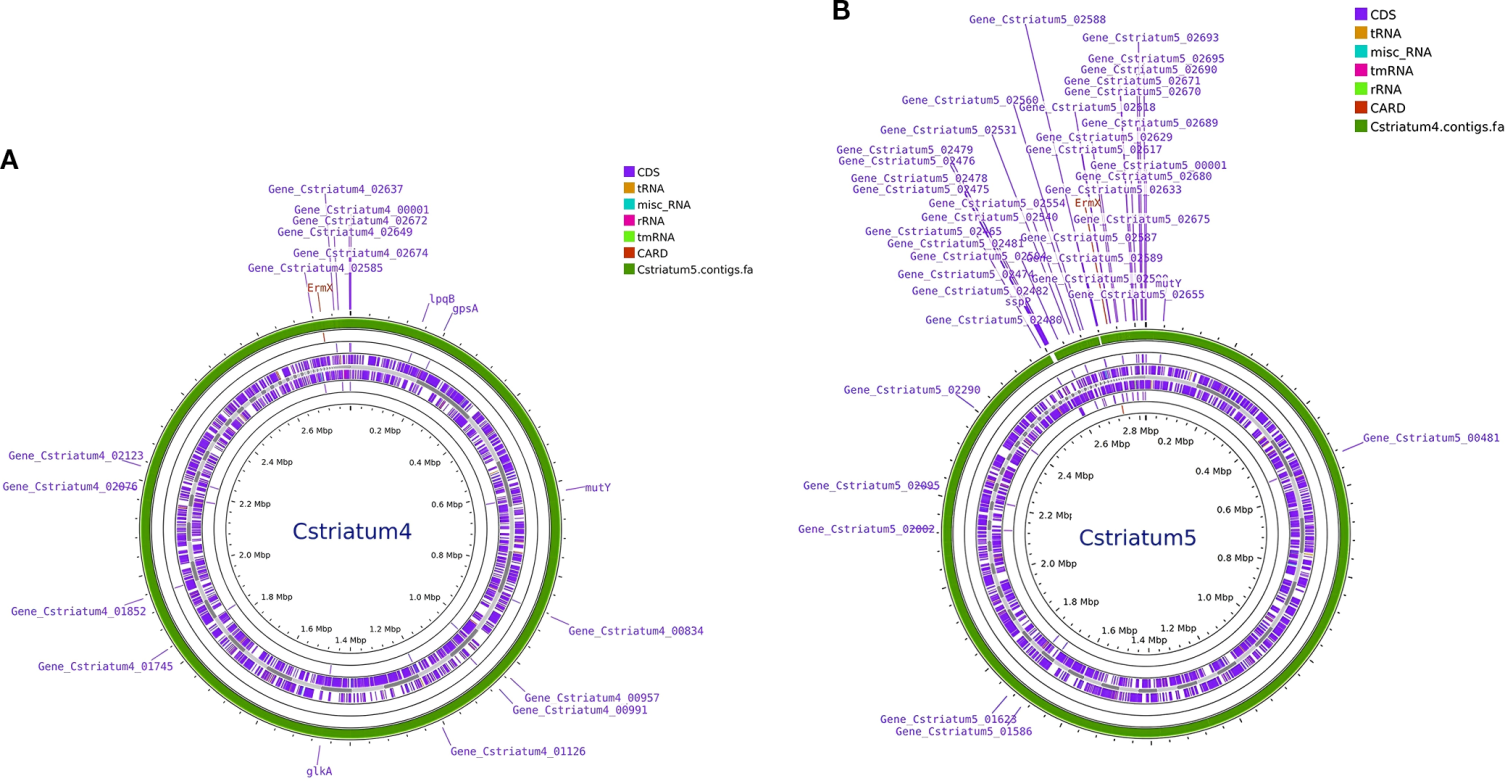
Genomic circular maps of *Corynebacterium striatum* isolates. **(A)** Cstriatum4; **(B)** Cstriatum5. Concentric rings depict coding sequences (CDSs), rRNA/tRNA loci, GC content and GC skew, and annotated antimicrobial resistance (AMR) genes. Both genomes show similar organization and GC features, consistent with the close relatedness of the two isolates.

**Figure 3:**
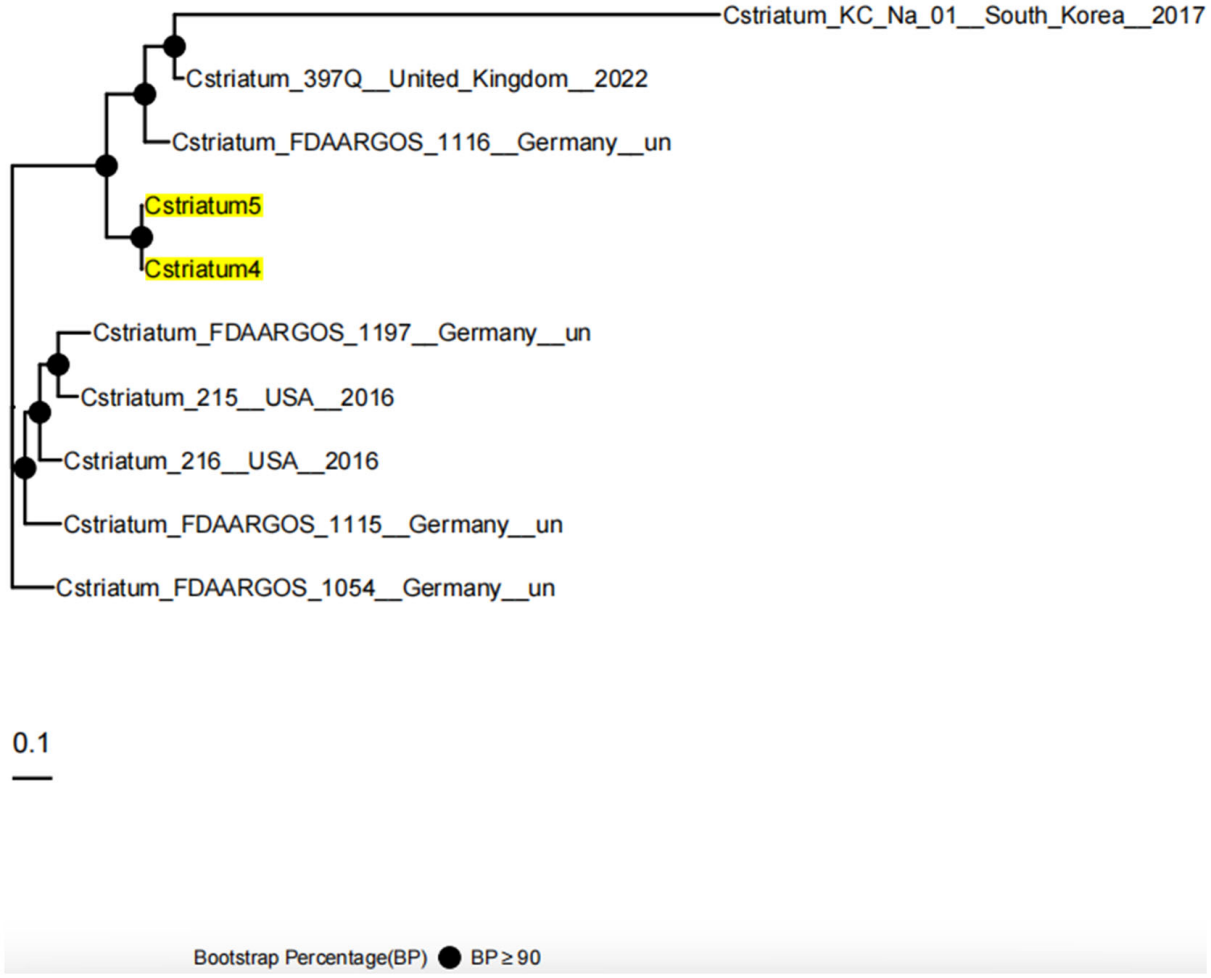
Core SNP phylogenetic tree of Cstriatum4 and Cstriatum5. The maximum-likelihood phylogenetic tree was constructed based on core SNP alignments to assess the genetic relationships between Cstriatum4, Cstriatum5, and reference genomes from multiple countries. Cstriatum4 and Cstriatum5 clustered together with high bootstrap support (BP ≥ 90), confirming their close genetic homology. The scale bar represents 0.1 nucleotide substitutions per site, and filled circles indicate nodes with strong bootstrap support.

Whole-genome sequencing was performed using the Illumina NovaSeq 6000 platform, and analysis was conducted with an integrated bioinformatics pipeline (including fastp, SPAdes, Prokka, among others). Comparative genomic analysis demonstrated that Cstriatum4 and Cstriatum5 were closely related, differing by only 54 SNPs, and shared an identical resistance gene profile (both carrying *ermX*). No canonical virulence genes were detected in the Virulence Factor Database (VFDB; http://www.mgc.ac.cn/VFs/), an international repository established and maintained by the Institute of Pathogen Biology, Chinese Academy of Medical Sciences. For phylogenetic analysis, assembled genomes were aligned to reference sequences using NUCmer (MUMmer v3.1) to define core genome regions. Core SNPs were then extracted and used to calculate evolutionary distances under a generalized time-reversible model implemented in FastTree v2.1.11. A maximum-likelihood phylogenetic tree was subsequently inferred using a combination of heuristic methods, including Neighbor-Joining and Minimum Evolution criteria, confirming that Cstriatum4 and Cstriatum5 belonged to the same cluster branch (**Figure 2**: Genomic circular maps; **Figure 3**: Core SNP phylogenetic tree).

### 2.4 Therapeutic intervention

**Figure 4:**
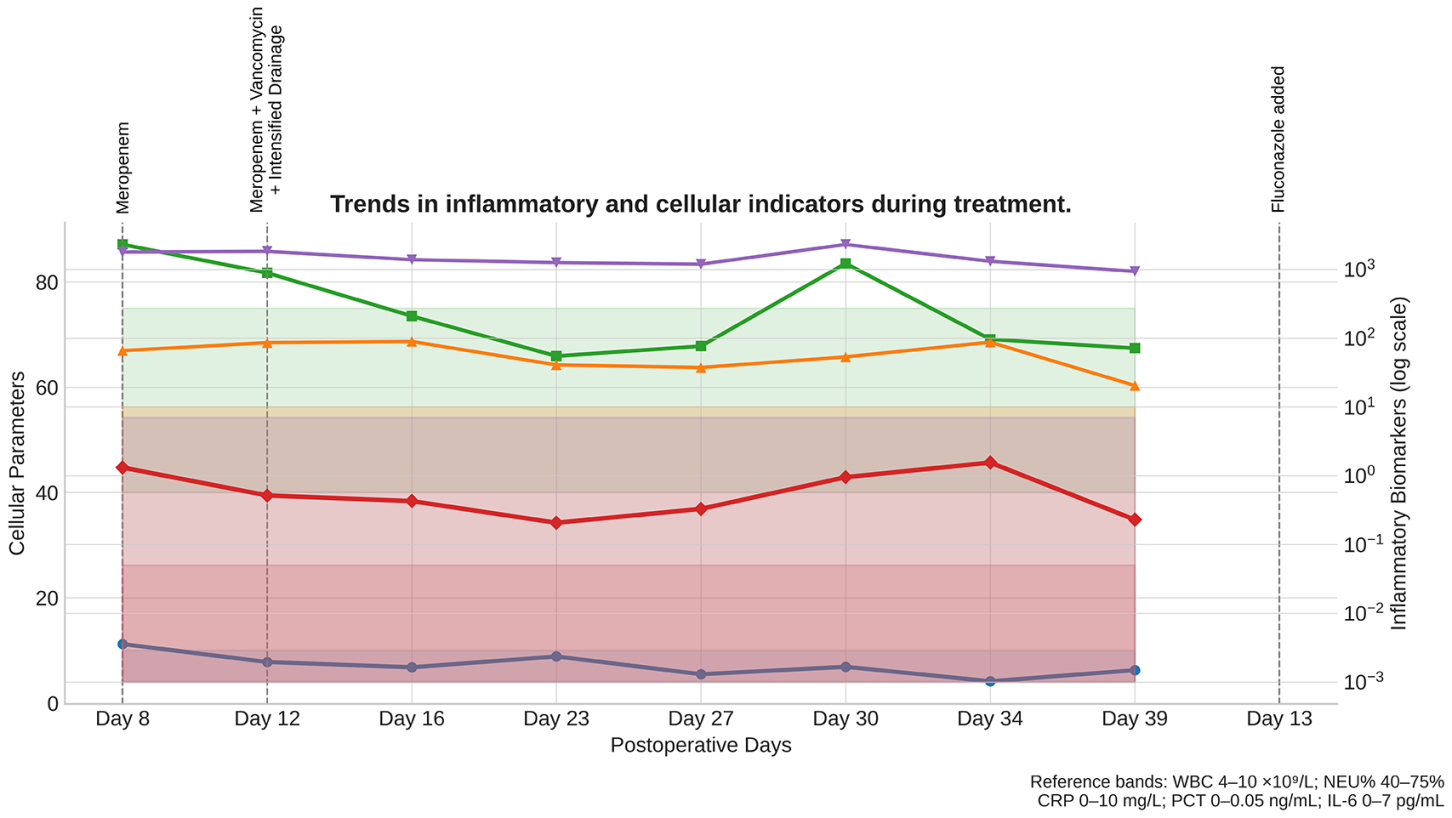
Trends in inflammatory and cellular indicators during treatment.

Following ICU admission, empirical meropenem (1 g every 8 hours, intravenous infusion) was initiated for antimicrobial coverage; however, inflammatory markers showed minimal improvement. After BALF culture results became available, antimicrobial therapy was adjusted to meropenem (1 g every 8 hours) plus vancomycin (1 g every 12 hours, intravenous infusion, with trough-level monitoring), accompanied by intensified peritoneal irrigation and drainage. Initial cultures of both BALF and ascitic fluid yielded pure growth of *Corynebacterium striatum*. In subsequent days, however, repeat cultures demonstrated mixed growth with Candida albicans, prompting the addition of short-term fluconazole (400 mg daily, intravenous infusion, for 5 days) for antifungal coverage. The abdominal drainage catheter was replaced on postoperative day 12 due to increased fluid accumulation, and was subsequently removed after imaging confirmed resolution of ascites and repeated cultures were negative. After 14 consecutive days of the adjusted regimen, PCT, IL-6, CRP, WBC, and NEU% all returned to normal ranges. Repeat abdominal ultrasonography revealed complete resolution of ascites, while chest CT demonstrated marked absorption of pulmonary lesions. The patient’s psychiatric symptoms stabilized, and he was successfully transferred back to the psychiatric ward (see **Figure 4**).

Serial measurements of WBC (white blood cell count), NEU% (neutrophil percentage), CRP (C-reactive protein), PCT (procalcitonin), and IL-6 (interleukin-6) are shown from ICU admission (Day 8) through recovery. Shaded reference bands indicate the normal ranges for each parameter. Dashed vertical lines mark the timing of major therapeutic interventions: Day 8, initiation of meropenem; Day 12, meropenem plus vancomycin with intensified drainage; and Day 13, addition of fluconazole. Progressive normalization of all parameters correlated with targeted antimicrobial therapy and supportive interventions, confirming effective infection control and resolution of systemic inflammation.

### 2.5 Follow-up and outcomes

After transfer from the ICU, the patient demonstrated a favorable clinical course. No recurrence of pleural effusion or abdominal fluid collections was observed, and subsequent respiratory specimens were consistently negative for *Corynebacterium striatum*. Follow-up evaluations revealed stable respiratory function, normalization of inflammatory markers, and no evidence of secondary infection. The patient required no additional antimicrobial therapy beyond the planned course, and recovery was maintained without relapse during the observation period.

## 3 Discussion

In this patient, highly homologous *Corynebacterium striatum* strains were isolated from both bronchoalveolar lavage fluid and ascitic fluid within a short postoperative interval. The infection course was closely linked to postoperative immunosuppression, compromised mucosal barriers, and ICU environmental exposure. It is plausible that during laparoscopic repair of the gastric perforation, the integrity of the peritoneal barrier was disrupted. Prolonged postoperative abdominal drainage further created favorable conditions for bacterial colonization and cross-contamination (6).In an ICU setting, characterized by high microbial load, airborne particles, and procedural contact, bacterial transfer across anatomical sites is possible. At the same time, endogenous translocation of colonizing *Corynebacterium striatum* and transmission via caregivers’ hands cannot be excluded. Given the limited evidence for long-term persistence of this organism on hospital surfaces, strict adherence to hand hygiene (7) and device management should be emphasized as the primary preventive measures, in line with international infection control guidelines, while targeted environmental surveillance may serve as a complementary tool in high-risk ICU scenarios to identify reservoirs during suspected outbreaks.

Antimicrobial susceptibility testing revealed that both Cstriatum4 and Cstriatum5 were broadly resistant to β-lactams and macrolides, retaining susceptibility only to glycopeptides and oxazolidinones, a profile consistent with previously reported ICU-derived isolates (8). The resistance phenotype may be attributable to the cell wall’s abundant arabinogalactan and corynemycolic acid, which not only fortify the bacterium against host antimicrobial peptides but also facilitate the development of dense biofilms under antimicrobial pressure (9). These biofilms enable long-term persistence in environments such as indwelling catheters and drainage fluids, greatly increasing the difficulty of eradication. In the present case, timely replacement of the abdominal drainage catheter on postoperative day 12 and its removal once ascites resolved and cultures became negative were critical steps in eliminating potential biofilm reservoirs, thereby supporting successful infection control.

Although no canonical virulence genes were detected in VFDB, several points support the pathogenic potential of the isolates. Both strains were cultured from normally sterile sites (BALF and ascitic fluid) at significant counts, and their recovery coincided with clear clinical signs of sepsis and peritonitis, accompanied by elevated inflammatory markers. The patient’s condition improved only after targeted antimicrobial therapy and drainage, strongly suggesting causation rather than colonization. In immunocompromised hosts, even organisms without classical virulence determinants may exploit weakened mucosal barriers and impaired neutrophil function to establish invasive infection. In the care of patients with psychiatric disorders, infection management and psychotropic medication adjustments must proceed in parallel to minimize drug–drug interactions and the adverse effects of immune suppression, thereby maximizing overall patient outcomes.

From an immunological standpoint, this patient’s advanced age, hypoalbuminemia, postoperative inflammatory stress, and prolonged clozapine therapy likely impaired innate immune defenses. Factors such as diminished neutrophil chemotaxis and phagocytic activity, inadequate complement activation, and reduced antimicrobial peptide secretion created favorable conditions for bacterial invasion (10). *Corynebacterium striatum* can engage TLR2, triggering macrophage release of pro-inflammatory mediators including IL-6 and TNF-α (11). In the context of immunosuppression, however, this inflammatory cascade often exhibits a “delayed onset with a higher peak,” a pattern that not only postpones infection control but also risks additional tissue injury (12).

The identification of multi-site infections caused by highly homologous strains carries important clinical implications. Such cases suggest that patients may be simultaneously exposed to two converging risks: dissemination of ICU environmental strains and downward migration of their own colonizing flora. For these patients, early in the disease course, it is critical to collect specimens from multiple sites—such as the respiratory tract and abdominal cavity—and perform both cultures and molecular homology analyses in parallel (13). This approach allows for the rapid determination of clonal relatedness and enables timely, targeted antimicrobial adjustments. For confirmed multidrug-resistant *Corynebacterium striatum* infections, empiric therapy should promptly include coverage with glycopeptides or linezolid, while avoiding unnecessary prolonged use of broad-spectrum β-lactams (14) to minimize resistance pressure. In parallel, stricter device management is essential: limiting the duration of endotracheal intubation and drainage tube placement, conducting daily assessments for device removal, and rigorously enforcing hand hygiene and aseptic techniques (15) to reduce opportunities for cross-contamination at the source.

Patients with psychiatric disorders present unique complexities in the diagnosis and management of infections. Clozapine, for example, can cause neutropenia or impair neutrophil function, significantly increasing the risk of severe infections; when combined with certain antimicrobials, such as meropenem, it may further interact at the level of metabolic pathways or immune regulation (16). Consequently, the management of such patients should include dynamic monitoring of immune-inflammatory markers such as white blood cell count, PCT, and IL-6, and, in close collaboration with psychiatrists, timely adjustment of antipsychotic dosages and administration schedules. Moreover, infectious delirium and systemic inflammatory responses can mask the improvement of pre-existing psychiatric symptoms—or even exacerbate them—leading to potential misjudgment of the clinical course (17). Therefore, a multidisciplinary team should track both mental status and infection control in parallel, ensuring that symptom overlap does not delay optimal treatment decisions.

## 4 Conclusion

This case also underscores the critical importance of infection control in the ICU setting. For patients with high-risk factors such as indwelling catheters, early assessment of extubation feasibility is essential, along with strict adherence to hand hygiene and aseptic protocols. Regular microbiological surveillance of the ICU environment should be implemented to reduce the colonization and spread of multidrug-resistant organisms. For confirmed MDR *Corynebacterium striatum* infections, prolonged reliance on broad-spectrum β-lactams should be avoided; instead, priority should be given to agents with proven activity—such as glycopeptides or oxazolidinones—with treatment duration and dosage adjusted promptly according to inflammatory marker trends.

## Data Availability

The datasets presented in this study can be found in online repositories. The names of the repository/repositories and accession number(s) can be found in the article/**Supplementary Material**.
